# Dose-Response Analysis Using R

**DOI:** 10.1371/journal.pone.0146021

**Published:** 2015-12-30

**Authors:** Christian Ritz, Florent Baty, Jens C. Streibig, Daniel Gerhard

**Affiliations:** 1 Department of Nutrition, Exercise and Sports, University of Copenhagen, Rolighedsvej 26, DK-1958 Frederiksberg C, Denmark; 2 Pneumology, Kantonsspital St. Gallen, Rorschacher Strasse 95, CH-9007 St. Gallen, Switzerland; 3 Department of Plant and Environmental Sciences, University of Copenhagen, Højbakkegård Allé 13, DK-2630 Taastrup, Denmark; 4 School of Mathematics and Statistics, University of Canterbury, Private Bag 4800, Christchurch 8140, New Zealand; University of Rochester, UNITED STATES

## Abstract

Dose-response analysis can be carried out using multi-purpose commercial statistical software, but except for a few special cases the analysis easily becomes cumbersome as relevant, non-standard output requires manual programming. The extension package *drc* for the statistical environment **R** provides a flexible and versatile infrastructure for dose-response analyses in general. The present version of the package, reflecting extensions and modifications over the last decade, provides a user-friendly interface to specify the model assumptions about the dose-response relationship and comes with a number of extractors for summarizing fitted models and carrying out inference on derived parameters. The aim of the present paper is to provide an overview of state-of-the-art dose-response analysis, both in terms of general concepts that have evolved and matured over the years and by means of concrete examples.

## Introduction

Dose-response analysis can be carried out using multi-purpose commercial statistical software. However, except for a few special cases the analysis easily becomes cumbersome as relevant, but non-standard output requires manual programming. Availability of specialized commercial statistical software for dose-response analysis is limited. We are aware of the commercial software GraphPad (http://www.graphpad.com) as well as a few standalone programmes (e.g., http://www.unistat.com and http://www.bioassay.de).

Over the last 20 years the open-source environment R [1] has developed into an extremely powerful statistical computing environment. The programming infrastructure has fuelled the development of highly sophisticated sub systems for more or less specialized statistical analyses within a number of scientific areas (e.g., the Bionconductor suite of packages: http://www.bioconductor.org). One such specialized sub system for analysis of dose-response data is provided through the add-on package *drc* [2]. There also exist a number of other **R** packages related to dose-response analysis: *DoseFinding* [3], *drfit* [4], *grofit* [5], *MCPMod* [6], and *nlstools* [7].

Originally, *drc* was developed to provide nonlinear model fitting for specialized analyses that were routinely carried out in weed science [2]. Subsequently the package has been modified and extended substantially, mostly in response to inquiries and questions from the user community. Meanwhile it has become a flexible and versatile package for dose-response analyses in general. Thus the present version of the package *drc* provides a user-friendly interface for specification of model assumptions about the dose-response relationship (including a flexible suite of built-in model functions) as well as for summarizing fitted models and making inference on derived parameters. The aim of the present paper is to provide an up-to-date account of state of the art for dose-response analysis as reflected in the functionality of *drc*.

## Dose-response models

Dose-response models are regression models where the independent variable is usually referred to as the dose or concentration whilst the dependent variable is usually referred to as response or effect.

### Types of responses

We define a dose (metameter) as any pre-specified amount of biological, chemical, or radiation stress eliciting a certain, well-defined response. Other kinds of exposure or stress could also be imagined, e.g., time elapsed in germination experiments. The dose is a non-negative quantity and it is often but not always assumed to be measured without error as is often the case in designed experiments [8].

Specifically, we define the response to a given dose as the quantification of a biologically relevant effect and as such it is subject to random variation. The most common type is a continuous response such as biomass, enzyme activity, or optical density. A binary or aggregated binary (binomial) response is also frequently used to describe results such as dead/alive, immobile/mobile, or present/absent [9]. The response may also be discrete as in number of events observed in a specific time interval such as number of juveniles, offspring, or roots [10]; this setting also encompassses Wadley’s problem considered in Example 6 in S1 File [11]. Dose-response curves may also be used to summarize experiments where the response is the event time, that is the time elapsed before some specific event is being observed [12, 13].

### Modelling the mean

The full specification of a statistical dose-response (regression) model involves specifying how the mean is described by a parametric function of dose as well as specifying assumptions about the distribution of the response.

We will focus on ways to model the mean trends through mostly s-shaped or related biphasic functions because these functions have in common that they reflect a basic understanding about the causal relationship between the dose and the response, e.g., when a dose increases the response monotonically decreases or increases one way or another towards minimum or maximum response limits, respectively. Consequently, these functions have turned out to be extremely versatile for describing various biological mechanisms involving model parameters that allow the interpretation of observed effects within a biologically plausible framework.

So we define dose-response models to be a collection of statistical models having a certain mean structure in common; this is not a strict mathematical definition, but rather a definition driven by applications. Consequently, dose-response models encompass a range of statistical models from nonlinear regression, generalized (non)linear regression, and parametric survival analysis.

Let *y* denote an observed response value, possibly aggregated in some way, corresponding to a dose value *x* ≥ 0. The values of *y* are often positive but may take arbitrary positive or negative values. Furthermore, we will assume that observation of *y* is subject to sampling variation, necessiating the specification of a statistical model describing the random variation. Specifically, we will focus on characterizing the mean of *y* (denoted *E*(*y*) below) in terms of a model function *f* that depends on the dose *x*:

$$\begin{array}{c} E(y)=f(x,\beta ). \end{array}$$ (1)

So, for a given dose *x*, the corresponding observed response values will be distributed around *f*(*x*, *β*). The function *f* is completely known as it reflects the assumed relationship between *x* and *y*, except for the values of the model parameters *β* = (*β*_1_, …, *β*_*p*_), which will be estimated from the data to obtain the best fitting function. The remaining distributional assumptions on *y* will depend on the type of response. For instance, for a continuous response the normal distribution is commonly assumed whereas for a binary or quantal response the binomial distribution is commonly assumed.

#### Built-in dose-response models

A large number of more or less well-known model functions are built-in in *drc* (see Table 1). These models are parameterized using a unified structure with a coefficient *b* denoting the steepness of the dose-response curve, *c*, *d* the lower and upper asymptotes or limits of the response, and, for some models, *e* the effective dose ED50.

**Table 1:** List of model functions and corresponding names of some of the most important built-in models available in *drc*.

Model type	Model function (*f*)	Function in *drc*
Generalized log-logistic	$c+\frac{d-c}{{\left(1+\exp(b(\log(x)-\log(e)))\right)}^{f}}$	llogistic()
Brain-Cousens	$c+\frac{d-c+fx}{1+\exp(b(\log(x)-\log(e)))}$	
Cedergreen-Ritz-Streibig	$c+\frac{d-c+f\exp(-1/\left(x^{\alpha }\right))}{1+\exp(b(\log(x)-\log(e)))}$	cedergreen()
*(*0 < *α* < 1 *is usually fixed in advance)*		
Log-logistic fractional polynomial	$c+\frac{d-c}{1+\exp(b{\left(\log(x+1)\right)}^{p_{1}}+e{\left(\log(x+1)\right)}^{p_{2}})}$	fplogistic()
Log-normal	*c*+(*d* − *c*)Φ(*b*(log(*x*) − log(*e*)))	lnormal()
*(*Φ: *distribution function for a normal distribution)*		
Weibull I	*c*+(*d* − *c*)exp( − exp(*b*(log(*x*) − log(*e*))))	weibull1()
Weibull II	*c*+(*d* − *c*)(1 − exp( − exp(*b*(log(*x*) − log(*e*)))))	weibull2()
Gamma	$c+\left(d-c\right)\tilde{\Gamma }\left(bx,e,1\right)$	gammadr()
*($\tilde{\Gamma }$: distribution function for a* Γ *distribution)*		
Multistage	*c*+(*d* − *c*)exp( − *b*_1_ − *b*_2_ *x* − *b*_3_ *x* ^2^)	multi2()
NEC	*c*+(*d* − *c*)exp(*b*(*x* − *e*)) for *x* > *e* and *d* otherwise	NEC.4()

By far the log-logistic models are the most used dose-response models [9]. The four-parameter log-logistic model corresponds to the model function:

$$\begin{array}{c} f\left(x,\left(b,c,d,e\right)\right)=c+\frac{d-c}{\left(1+\exp(b(\log(x)-\log(e)))\right)} \end{array}$$ (2)

Two slightly different parameterizations are available: one where *ED*50 is a model parameter, that is *e* in Eq (2), and another where the logarithm of *ED*50 denoted by $\tilde{e}$, say, is a model parameter as in the following Eq (3):

$$\begin{array}{c} f\left(x,\left(b,c,d,\tilde{e}\right)\right)=c+\frac{d-c}{\left(1+\exp\left(b\left(\log\left(x\right)-\tilde{e}\right)\right)\right)} \end{array}$$ (3)

This second version of the four-parameter log-logistic model may be preferred for dose-response analysis involving very small datasets (<15–20) where a normally distributed parameter estimate may more reasonably be assumed on a logarithm-transformed dose scale than the original dose scale.

Model functions derived from the log-logistic model include the generalized five-parameter log-logistic model, which results in an asymmetric dose-response curve [14], the five-parameter baroflex model (with two slope parameters *b*) [15], and the non-monotonous hormesis models [16, 17]. Log-logistic type fractional polynomial dose-response models were introduced for a binomial response [18]. These models have been implemented in *drc* in novel, extended four-parameter versions that are also equally suitable for describing a dose-response curve for a continuous response. Biphastic functions obtained as the sum of two four-parameter log-logistic models may also be fitted using *drc* [19]. Recently, other types of biphasic dose-response models were proposed in the context of biosensors [20].

Log-normal models, which result in dose-responses curves very similar to curves obtained from the corresponding log-logistic models, and two types of asymmetric Weibull models are also available in *drc*. In passing we note that there is a close link between the two-parameter log-logistic, log-normal, and Weibull type I models available in *drc* (where only the two parameters *b* and *e* are not fixed) and the log-logistic, log-normal, and Weibull models available in the package *survival* [21]. Generalized four- and five-parameter versions of the gamma and (quadratic) multistage models, respectively, are also implemented [22]. Another built-in model is the so-called “no effect concentration” (NEC) threshold model [23].

Most of these functions are scale invariant in the sense that the magnitude of doses is accommodated by the model itself through the parameter *b*. For instance, for log-logistic functions the slope parameter *b* acts as a scaling factor, centering doses around 1. In contrast the Brain-Cousens and Cedergreen-Ritz-Streibig models are sensitive to the magnitudes of the doses, which may need to be manually up- or downscaled appropriately prior to model fitting. Moreover, some of these functions may be used for fitting both decreasing and increasing dose-response curves. One exception is the hormesis models, which can only describe decreasing trends, i.e., they are so-called inverse j-shaped hormesis models. However, modified functions for u-shaped hormesis models for increasing dose-response curves are also available [24]. It is also possible to define your own model function [25].

#### Hierarchical model structure

A key distinguishing feature of *drc* is the hierarchical structure of the built-in model functions, which provides a convenient way to specify special cases obtained by fixing one or more parameters *a priori* at certain given values. These parameters will not be estimated from the data, but will be kept fixed at the specified values. In practice such special cases occur quite frequently. For instance, various special cases may be derived from the general four-parameter log-logistic model function [26] (see also Example 4 in S1 File).

All built-in model functions accept the argument fixed, which is used for fixing parameters at given values. For instance, log-logistic models with varying number of model parameters may be specified as follows:

- Fixing *f* = 1:
  $$\begin{array}{c} c+\frac{d-c}{1+\exp(b(\log(x)-\log(e)))} \end{array}$$
  In *drc*: LL.5(fixed = c(NA, NA, NA, NA, 1)) or abbreviated LL.4()
- Fixing both *f* = 1 and *c* = 0:
  $$\begin{array}{c} \frac{d}{1+\exp(b(\log(x)-\log(e)))} \end{array}$$
  In *drc*: LL.4(fixed = c(NA, 0, NA, NA)) or abbreviated LL.3()
- Fixing *f* = 1, *c* = 0, and *d* = 1:
  $$\begin{array}{c} \frac{1}{1+\exp(b(\log(x)-\log(e)))} \end{array}$$
  In *drc*: LL.4(fixed = c(NA, 0, 1, NA)) or abbreviated LL.2()

Note that NAs are used to indicate that parameters have to be estimated from the data. Note also that the order of model parameters is *b*, *c*, *d*, *e*, *f*.

Fixing model parameters at a priori chosen values should not be confused with constrained optimisation where the ranges of some parameters are constrained, but they are still being estimated.

#### Models for several dose-response curves

Some experiments result in data corresponding to several dose-response curves. One example is fixed-ratio ray design mixture toxicity experiments where data consist of a number of dose-response curves corresponding to the different mixture ratios applied [27]. Such data may be modelled using joint models where individual dose-response curves are still assumed but with constraints on parameters across curves. Recently, it was described how to use *drc* for fitting such models [28].

For complex, hierarchical dose-response experiments it may be advantageous to fit separate models initially. In a second step relevant parameter estimates extracted from these separate model fits are treated as response in another statistical analysis: one such example of a two-step approach used *drc* initially and subsequently the package *flexmix* [29].

## Estimation procedures

In *drc* estimation of parameters is based on the maximum likelihood principle, which under the assumption of normally distributed response values simplifies to nonlinear least squares. Estimation of the parameters in dose-response models or any derived parameters will use all observations but for specific parameters observations may still contribute in an unequal way, e.g., some parameters will be more determined by observations in one of the tails than other parameters.

The model fitting function in *drc* is called drm(). Contrary to most other statistical software programmes for dose-response analysis the dose 0 is left *as is* during the estimation using drm(), meaning that no value (such as 0.1 or 0.01) is added to the dose to be able to calculate values for dose 0. This is achieved by incorporating in the implementation that the model functions are mathematically well-defined also for dose 0.

### Nonlinear least squares

Assume that *x*_1_, …, *x*_*n*_ are the dose values and *y*_1_, …, *y*_*n*_ the corresponding observed response values. The nonlinear least squares estimates are obtained by minimizing the following sum of squares:

$$\begin{array}{c} \sum_{i=1}^{n}w_{i}^{2}{\left\{y_{i}-f\left(x_{i},\beta \right)\right\}}^{2} \end{array}$$ (4)

where *w*_*i*_’s are user-specified weights (often left unspecified, i.e., equal to 1). In drm() weights are specified through the argument weights and they should be on the same scale as the response, e.g., expressed as standard deviations and not empirical variances. Weights may be used for addressing variance heterogeneity in the response. However, the transform-both-sides approach should be preferred over using often very imprecisely determined weights (see the next subsection).

Eq (4) has to be solved numerically in an iterative manner. One approach is to use iteratively weighted least squares as is done in nls(), which is part of standard installation in **R** [30]. Another approach is to use a general-purpose minimizer directly, as in drm() where optim() is used in combination with some pre-scaling of parameters. In our experience drm() is more robust than nls(); lack of robustness of nls() has also been pointed out previously [31].

The estimated variance-covariance of the parameter estimates ($\text{cov}(\hat{\beta })$) is obtained as the scaled inverse of the observed information matrix, which consists of second-order partial derivatives of *f* w.r.t. the model parameters *β*_1_, …, *β*_*p*_:

$$\begin{array}{c} \text{cov}\left(\hat{\beta }\right)={\hat{\sigma }}^{2}{\left({\left\{\frac{\partial^{2}f}{\partial \beta_{p_{1}}\partial \beta_{p_{2}}}\right\}}_{p_{1},p_{2}\in \left\{1,\ldots ,p\right\}}\right)}^{-1}={\hat{\sigma }}^{2}{\left(\frac{\partial^{2}f}{\partial \beta_{p_{1}}\partial \beta_{p_{2}}}\right)}^{-1} \end{array}$$ (5)

The scaling factor $\hat{\sigma }$ is the residual standard error, which is estimated in the same way as in linear regression based on residuals. The observed information matrix (“hessian”) in Eq (5) is approximated numerically in optim() upon convergence. However, it is not even a requirement that the inverse is available: if it is not then NAs are returned for standard errors; see Example 1 in S1 File.

### Transform-both-sides approach

The transform-both-sides approach implies that both the response and the nonlinear model function are shifted by a constant *C* and then transformed by a suitable function *g*_λ_ [32]. The resulting model for the mean of the transformed response looks like this:

$$\begin{array}{c} E\left\{g_{\lambda }\left(y+C\right)\right\}=g_{\lambda }\left(f\left(x,\left(\beta_{1},\ldots ,\beta_{p}\right)\right)+C\right) \end{array}$$ (6)

with the transformed response on the left-hand side and the transformed model function on the right-hand side. Usually the function *g*_λ_ is taken to be the Box-Cox transformation *g*_λ_(*y*) = (*y* ^λ^ − 1)/λ for some suitable choice of λ∈R. The value λ = 1 implies no transformation whilst λ = 0 corresponds to the logarithm transformation. All other values of λ correspond to power transformations. It is noteworthy that the Box-Cox transformation may alleviate variance heterogeneity and some skewness in the distribution of the response and thus recover a normal distribution, but it may not remedy other problems with the distributional assumptions such as counts observed with ties [10].

The package *drc* provides a method boxcox, which has been implemented in much the same way as the corresponding method for linear models available in the package *MASS* [33]. There is, however, a choice between a profiling approach as used for linear models in *MASS* or a more robust analysis of variance (ANOVA) approach where the optimal λ is estimated from a more general ANOVA model, i.e., a linear model, and not from the specified dose-response model; the latter requires replicate observations for at least some doses.

Manual specification of the transformation is also possible through the arguments bcVal and bcAdd, which correspond to λ and *C* in Eq (6), respectively. Using a specific transformation (i.e., a specific value of λ and possibly *C*) based on previous experience should be preferred over using a data-driven choice.

### Maximum likelihood estimation

In case of maximum likelihood estimation the following expression has to be maximized in terms of *β* values in order to obtain parameter estimates:

$$\begin{array}{c} \text{log}\;\text{likelihood}=\sum_{i=1}^{n}l\left\{y_{i},f\left(x_{i},\beta \right)\right\} \end{array}$$ (7)

where *l* denotes the logarithm-transformed likelihood function for a single measurement or observation. The likelihood in turn depends on the distributional assumptions made for the dose-response model. The estimated variance-covariance of the parameter estimates is found as the inverse of the observed information matrix [34]:

$$\begin{array}{c} \text{cov}\left(\hat{\beta }\right)={\left(\frac{\partial^{2}l}{\partial \beta_{p_{1}}\partial \beta_{p_{2}}}\right)}^{-1} \end{array}$$ (8)

Maximum likelihood estimation is used for fitting dose-response models to bionomial, count, and event-time data, including models for binomial data where natural immunity and/or natural mortality is present [11].

### Robust estimation

One way to handle observations that seem to be extreme or deviating in one way or another as compared to the bulk of the observations is to apply a robust estimation procedure, which will weigh down the influence of such observations.

Robust nonlinear regression is available through the function nlrob() in the **R** package *robustbase*. This function relies on nls() through an iterated weighted least squares approach. However, no self starter functionality is currently available. The commercial software GraphPad also has some limited functionality for robust nonlinear regression, but the resulting model fits are provided without standard errors of parameter estimates [35].

In *drc* robust dose-response analysis with a continuous response is available using the same models as considered in the case of robust linear regression [33], except for the model based on Hampel’s *ψ*, which is currently not implemented. However, it may be difficult to fit such robust nonlinear models unless fairly accurate starting values for the model parameters are provided.

The estimated “information”-type variance-covariance has the following form [36, 37]:

$$\begin{array}{c} \text{cov}\left(\hat{\beta }\right)={\tilde{\sigma }}^{2}{\left(\frac{\partial^{2}\rho }{\partial \beta_{p_{1}}\partial \beta_{p_{2}}}\right)}^{-1} \end{array}$$

where the function *ρ* controls the influence of observations on the estimation procedure (e.g., *ρ*(*y*) = *y* ^2^ corresponds to ordinary nonlinear least squares estimation). Once parameter estimates have been obtained all methods and extractors available in *drc* may be used as if the model fit had been obtained using maximum likelihood estimation.

### Constrained estimation

By default unconstrained estimation is carried out by drm() although for most models there are actually constraints on at least some of the model parameters, e.g., ED50 needs to be positive. Likewise, there may also be constraints needed to ensure uniqueness of the model fits, e.g., the slope parameter *b* should be negative for an increasing dose-response relationship to ensure the interpretation that *c* is the lower asymptote and *d* the upper asymptote. In practice such constraints are indirectly enforced through the appropriate choice of starting values.

However, in principle any of the aforementioned estimation procedures may be combined with the use of constraints where the range of one or more parameters is restricted, e.g., to certain intervals by setting lower and upper bounds (so-called box constraints) that are different from −∞ and ∞, respectively. This is possible with drm() using the arguments lowerl and upperl, which, if specified, will invoke constrained optimisation through the method “L-BFGS-B” in optim().

### Self starter functions

The estimation procedures introduced in this section are iterative and to ensure convergence towards optimal parameter estimates they need to be initiated through the provision of starting values for the model parameters. The choice of starting values may crucially affect whether convergence is eventually achieved. Thus, availability of good starting values facilitate parameter estimation in nonlinear models. Starting values may be obtained either by using parameter estimates previously reported for similar experiments or, in a data-driven way, by using the dose-response data themselves to elicit relevant information. For instance, within weed science, routine calculation of starting values for log-logistic models from simple linear regression models of transformed responses dates back at least to [38], utilizing extending linearization techniques previously used for probit analysis [39].

The flexibility of **R** has made it possible to implement so-called self starter functions that return data-driven starting values for the model parameters. In **R** self starter functions are available for the function nls(). In *drc* the above-mentioned data-driven linearization technique has been extended to other types of dose-response models and it is now available for all built-in models. It is the default setting, which may, however, be overruled by supplying starting values manually through the argument start. The function getInitial() may be used to obtain the starting values that were actually used for obtaining a particular model fit.

More sophisticated self starter functions have been proposed [40], but so far they have not implemented in *drc* except for some log-logistics models.

### Choice of model

Ideally, either a single dose-response model is fitted based on established knowledge or a number of plausible candidate models are identified and a model-averaging approach is adopted. However, in reality a decision-tree approach is typically used to find the best-fitting model among several candidates. For this approach the function mselect() may be useful as it returns values for information criteria or, if models are nested, p-values from lack-of-fit/goodness-of-fit tests as implemented in the function modelFit().

## Obtaining relevant parameter estimates

The methods summary() and coef() may be used to retrieve the estimated model parameters with and without the corresponding naive standard errors and derived z- or t-test statistics and p-values. Standard errors, which are robust against misspecification of the distributional assumptions, may easily be obtained by means of the packages *lmtest* and *sandwich* as shown in Example 2 in S1 File [41, 42].

By default the p-values reported in the summary() output are unadjusted. However, adjusted p-values controlling the family-wise error rate may be obtained using the function glht() in the package *multcomp*, assuming that test statistics jointly follow a multivariate normal or *t* distribution [43]. Note that it is even possible to use robust standard errors and then carry out simultaneous inference.

In case several dose-response curves are fitted simultaneously the function compParm() is useful for comparisons between groups *within* a single model parameter; see Example 3 in S1 File. Occassionally, global *F* tests and, in general, chi-square likelihood ratio tests may be useful for assessing if a number of parameters could be identical, e.g., several ED50 values. Specifically, these tests need two dose-response model fits such that one fit should be nested within the other. The model fits may then be compared using the anova() method; see again Example 3 in S1 File. A related convenience function is noEffect(), which may be used to test if there is any dose-response relationship at all, i.e., comparing the dose-response model to a model where the response is on average constant and hence not changing with dose.

Next, we will consider how to estimate various derived parameters that are functions of the model parameters and that are often of key interest in dose-response analysis.

### Inverse regression

Estimating doses corresponding to specific response levels is often of particular interest in dose-response analysis. Such estimates are obtained by solving an inverse regression problem, which may be approached either through after-fitting or re-parameterization. After-fitting means solving the inverse regression problem *after* having fitted the model. For some models the solution may be derived explicitly (e.g., log-logistic models [2, 44]) whereas for other models only numerical solutions are available (e.g., hormesis models [17]). After-fitting relies on the so-called delta method, which enables the calculation of approximate standard errors for derived parameter estimates [45]. Reparameterization means fitting the model using a reparameterized model function where the solution is an actual model parameter.

A practical implication of the after-fitting approach is that it suffices to fit a dose-response model once, in a parameterization that has proven to be the most stable for estimation. In our opinion, after-fitting provides a major improvement over the often quite instable re-parameterization approaches still being used with other statistical software programmes [46].

#### Estimating effective doses

The most prominent example of a derived parameter obtained using inverse regression is the dose that results in a halfway reduction between the lower and upper limits of the dose-response curve *f* as doses approach infinity and 0, respectively. This parameter is often denoted *ED*50 or *EC*50 for continuous responses, *LD*50 or *LC*50 for binomial responses, and *T*_50_ for event-time responses. We will use the term *effective dose*.

For a decreasing mean function *f* the effective dose for 0 < *α* < 1 (*ED*100*α*) is defined as the solution to the following equation:

$$\begin{array}{c} f\left(ED100\alpha ,\beta \right)=\left(1-\alpha \right)\lim_{x\to 0}f\left(x,\beta \right)+\alpha \lim_{x\to \infty }f\left(x,\beta \right)=\left(1-\alpha \right)c+\alpha d \end{array}$$ (9)

Note that these limits are well-defined and finite for dose-response models (for most models in *drc* they correspond to the parameters *d* and *c*, respectively).

The resulting effective dose is a relative quantity, defined in terms of a percentage reduction. For instance, *ED*50 (*α* = 0.50) is the dose resulting in a 50% reduction in the average response relative to the lower and upper limits of *f*. Such relative effective doses are mostly suitable for continuous responses. For increasing *α* the corresponding *ED*100*α* will be increasing as a consequence of *f* being a decreasing function.

For binomial responses absolute effective doses referring to the entire probability scale [0, 1], which not necessarily coincide with the lower and upper limits of the estimated dose-response curve, are usually more relevant. In general, an absolute effective dose *EDy*_0_ corresponding to an average response *y*_0_ may be defined as the solution to the following equation:

$$\begin{array}{c} f\left(EDy_{0},\beta \right)=y_{0} \end{array}$$ (10)

An absolute effective dose for *y*_0_ ∈]*c*, *d*[ may always be calculated as some relative effective dose for suitably derived *α* value. In some cases this approach will involve parameter estimates for the lower and upper limits, but at present the variation in these estimates will not be propagated to the estimated effective dose.

Estimated effective doses are obtained by inserting parameter estimates and solve Eq (9) w.r.t. the derived parameter *ED*100*α*. In *drc* the function ED() will calculate estimated effective doses. Estimation of ED values is shown in Example 2 and 5 in S1 File. In ED() the argument type controls the type of effective dose being calculated: relative (default) or absolute. Note also that for hormesis models effective doses may also be meaningfully defined for some *α* < 0 (by setting the argument bound = TRUE in ED()). Model-averaged estimated ED values may be obtained through the function maED() [47].

The ratio of effective doses at levels 100*α*_1_ and 100*α*_2_, respectively, of two fitted dose-response curves denoted A and B, say, is defined as follows:

$$\begin{array}{c} \rho \left(\alpha_{1},\alpha_{2}\right)=\frac{ED_{A}100\alpha_{1}}{ED_{B}100\alpha_{2}},\;0<\alpha_{1},\alpha_{2}<1 \end{array}$$

For the important special case where *α*_1_ = *α*_2_ the ratio is referred to as the relative potency [11], and it is interpreted as a measure for quantifying the strength of one substance over another, i.e., a pairwise comparison based on a ratio. The term selectivity index is also sometimes used [48]. In general, the ratio may be interpreted as the order of magnitude of the window between harmful and safe for appropriate choices of *α*_1_ and *α*_2_, e.g., 0.1 an 0.9. In some special cases (“parallelism”) the relative potency is constant between two dose-response curves for all values satisfying *α*_1_ = *α*_2_. More often it will change with the value of *α*_1_ = *α*_2_ and this may be investigated using the function relpot() [49].

In *drc* the extractor function EDcomp() may be used for obtaining estimated relative potencies or differences between effective doses. By default the corresponding standard errors are derived using the delta method and confidence intervals are based on these standard errors (indicated through the argument interval = “delta”) in combination with t- or z-value (percentile in an appropriate t- or normal distribution). Alternatively, confidence intervals based on the approach of Fieller may be used (interval = “fieller”). For the special case of the log-logistic model where *log*(*ED*50) is a model parameter ($\tilde{e}$) confidence intervals on the original dose scale may be retrieved through back transformation (interval = “fls”).

#### Benchmark dose estimation

For any type of response (e.g., binomial and continuous) benchmark dose (BMD) estimation may be reduced to a special case of estimating effective doses [50]. The key implication is that dose-response modelling (in all kinds of extensions and modifications) will allow subsequent BMD estimation, opening up for using the BMD concept much more broadly than what is currently done. Specifically, there is no longer a need for special modelling approaches that differ depending on the type of response. The **R** package *bmd* provides BMD estimation for single dose-response curves fitted using drm(), using either added or excess risk definitions. Finally, simultaneous inference for multiple BMD levels is also possible [51].

### Prediction

There are a number of functions available in *drc* for obtaining predicted values from a dose-response model fit. Notably, there are the general-purpose methods fitted() and predict(). The latter may also provide confidence or prediction intervals (controlled through the argument type). The function backfit() may be used for obtaining the estimated doses that correspond to the observed average response at each dose.

The predict() method is useful for obtaining data for construction of plots using the traditional graphical functionality in **R**. In particular, the plot() method may be used to show the original or summarized data together with the fitted dose-response curve(s) superimposed. Such plots are often shown in articles as they are useful for assessing model fit and they could be used together with residual and normal probability plots. By default plot() uses a logarithmic dose axis, which may be switched off with the argument log = “”. The argument broken allows dose 0 also to be shown in plot together with the logarithmic axis. Normalization of the response relative to the control (dose 0) may be achieved using normal = TRUE and possibly the argument normref for setting the reference, which is by default 1, as suggested by [52]. This normalization is based on the model fit and it does not involve modification of the original data.

For more sophisticated plots, however, we suggest to use the package *ggplot2* as shown in Example 2 in S1 File.

## Discussion

We have described the key functionality presently available in the **R** extension package *drc*. An important feature is that the package *drc* may be used in combination with other extension packages in **R** such as *ggplot2* and *multcomp*, enhancing the flexibility and usefulness of the package.

There is still room for extending the capabilities of the built-in models and for incorporating methods for handling additional types of responses such as ordinal responses, e.g., obtained from the assessment of severity of damage on weeds after applying a herbicide (a score between 0 and 10). It could also be useful to have beta-binomial dose-response models implemented. The functionality for benchmark dose estimation could also be extended. Likewise robust dose-response analysis for other types of responses could be a useful extension.

It would also be useful to implement alternative ways of estimating the uncertainty of (derived) parameter estimates, e.g., bootstrap and profiling approaches or inversion of confidence/prediction intervals.

The presented framework has also some limitations. Notably, we have throughout the entire paper assumed that responses are mutually independent. Certain correlation structures may readily be implemented within the framework of *drc*, e.g., correlation that is a function of the dose-response model function [53]. More generally, for continuous responses the package *medrc* extends many of the capabilities of *drc* to correlated dose-response data fitted through nonlinear mixed-effects regression models [54]. Specifically *medrc* utilizes the capabilities of the package *nlme* [55], but *medrc* allows both estimation of conditional and population-based ED levels; the latter has not been available for dose-response analysis previously.

## Supporting Information

S1 File Five illustrative examples of dose-response modelling. (PDF)
