# Supplementary material for: Dose-Response Analysis Using R
Source: PLoS One. 2015 Dec 30;10(12):e0146021. doi: 10.1371/journal.pone.0146021 (PMC4696819; doi:10.1371/journal.pone.0146021)
Supplement: S1 File — (PDF) [file pone.0146021.s001.pdf]

Dose-response Analysis Using R

Supplementary information S1

Christian Ritz, Florent Baty, Jens C. Streibig, and Daniel Gerhard

Illustrative examples

The datasets used in the following examples are all available as built-in datasets in the package *drc* version 2.6-10.

Example 1: A cautionary example

We start out fitting a five-parameter log-logistic dose-response model to an artificial dataset consisting of two dose values and corresponding response values.

```
dose <- 1:2
resp <- rnorm(length(dose))
# no need to set the seed for the random number generation!
resp
```

```
## [1] 0.1934897 1.3011434
```

```
library(drc)
m <- drm(resp ~ dose, fct = LL.5())
summary(m)
```

```
##
## Model fitted: Generalized log-logistic (ED50 as parameter) (5 parms)
##
## Parameter estimates:
##
##                   Estimate  Std. Error     t-value p-value
## b:(Intercept) -1.9934e+01  2.0115e-11 -9.9101e+11      NA
## c:(Intercept)  1.9238e-01          NA          NA      NA
## d:(Intercept)  1.3023e+00          NA          NA      NA
## e:(Intercept)  1.4142e+00  4.1268e-13  3.4269e+12      NA
## f:(Intercept)  1.0000e+00  3.5510e-12  2.8161e+11      NA
##
## Residual standard error:
##
##  NaN (-3 degrees of freedom)
##
## Warning: Too complex model fitted as df<1
```

Standard errors close to 0 reflect that the dose-response model fitted the two points perfectly as is also seen in the figure, just like a straight line will always fit exactly through two points as well. However, both these examples illustrate overfitting: the statistical models are too complex in view of the limited available data.

```
plot(m)
```

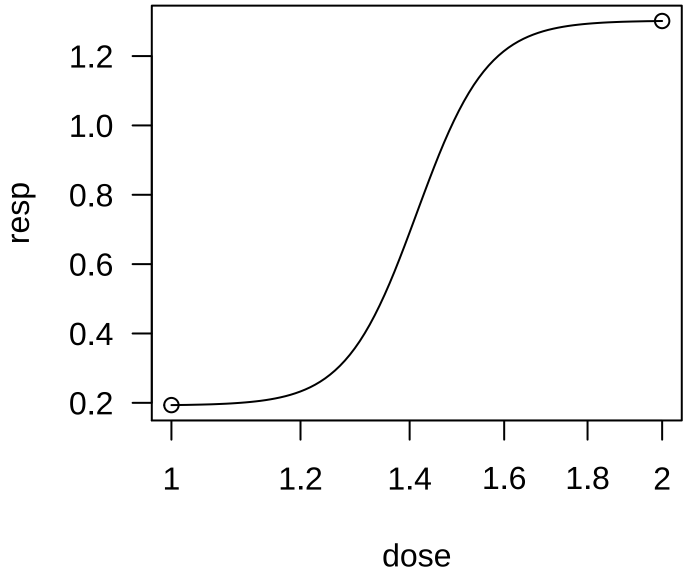

It can be shown in general that the estimated lower and upper asymptotes will invariably be equal to the two observed response values. Likewise the parameters b and f will invariably be estimated to be $\pm\infty$ (depending on whether there is a decrease or increase between the two points) and 1, respectively. The estimate of the parameter e will be equal to the geometric mean of the two dose values, which in this example is $\sqrt{2} \approx 1.4142$ (for logistic models the estimate will be the arithmetic mean). This estimate of e coincides with the binomial method estimate (Stephan, 1977; Environment Canada, 2005).

Example 2: Continuous response: one dose-response curve

We consider dose-response data on the effect of a herbicide in growth of perennial ryegrass (Inderjit *et al.*, 2002).

```
ryegrass.LL.4 <- drm(rootl ~ conc, data = ryegrass, fct = LL.3())
summary(ryegrass.LL.4)

##
## Model fitted: Log-logistic (ED50 as parameter) with lower limit at 0 (3 parms)
##
## Parameter estimates:
##
##                Estimate Std. Error  t-value p-value
## b:(Intercept)  2.47033    0.34168  7.22987       0
## d:(Intercept)  7.85543    0.20438 38.43517       0
## e:(Intercept)  3.26336    0.19641 16.61536       0
##
## Residual standard error:
##
##  0.5615802 (21 degrees of freedom)

#shMat <- matrix(c(1,0.5,8,3),24, 4, byrow=TRUE)
#ryegrass.LL.4x <- drm(rootl~conc, data=ryegrass, fct=LL.4(), pshifts=shMat, start=c(0,0,0,0))
#summary(ryegrass.LL.4x)
```

Next, we use the **R** packages *lmtest* and *sandwich* to obtain robust standard errors to address the fact that some variance heterogeneity is present.

```
library(sandwich)
library(lmtest)
coeftest(ryegrass.LL.4, vcov = sandwich)

##
## t test of coefficients:
##
##                Estimate Std. Error t value  Pr(>|t|)
## b:(Intercept)  2.47033    0.29238   8.449 3.394e-08 ***
## d:(Intercept)  7.85543    0.15397  51.020 < 2.2e-16 ***
## e:(Intercept)  3.26336    0.26572  12.281 4.744e-11 ***
## ---
## Signif. codes:  0 '***' 0.001 '**' 0.01 '*' 0.05 '.' 0.1 ' ' 1
```

Simultaneous inference is also possible through the use of the function `glht()` in the **R** package *multcomp*:

```
library(multcomp)
summary(glht(ryegrass.LL.4))

##
##   Simultaneous Tests for General Linear Hypotheses
##
## Fit: drm(formula = rootl ~ conc, data = ryegrass, fct = LL.3())
##
## Linear Hypotheses:
```

```
##                      Estimate Std. Error z value Pr(>|z|)
## b:(Intercept) == 0   2.4703     0.3417    7.23   <1e-10 ***
## d:(Intercept) == 0   7.8554     0.2044   38.44   <1e-10 ***
## e:(Intercept) == 0   3.2634     0.1964   16.61   <1e-10 ***
## ---
## Signif. codes:  0 '***' 0.001 '**' 0.01 '*' 0.05 '.' 0.1 ' ' 1
## (Adjusted p values reported -- single-step method)
```

Estimating effective doses ED5, ED10, and ED50 is accomplished using `ED()`:

```
ED(ryegrass.LL.4, c(5, 10, 50), interval = "delta")

##
## Estimated effective doses
## (Delta method-based confidence interval(s))
##
##         Estimate Std. Error   Lower  Upper
## e:1:5    0.99088    0.17808 0.62053 1.3612
## e:1:10   1.34086    0.18904 0.94773 1.7340
## e:1:50   3.26336    0.19641 2.85491 3.6718
```

where 95% confidence intervals are obtained using the delta method.

```
plot(ryegrass.LL.4, broken = TRUE, type = "all",
     xlab = "Ferulic acid (mM)", xlim = c(0, 100),
     ylab = "Root length (cm)")
```

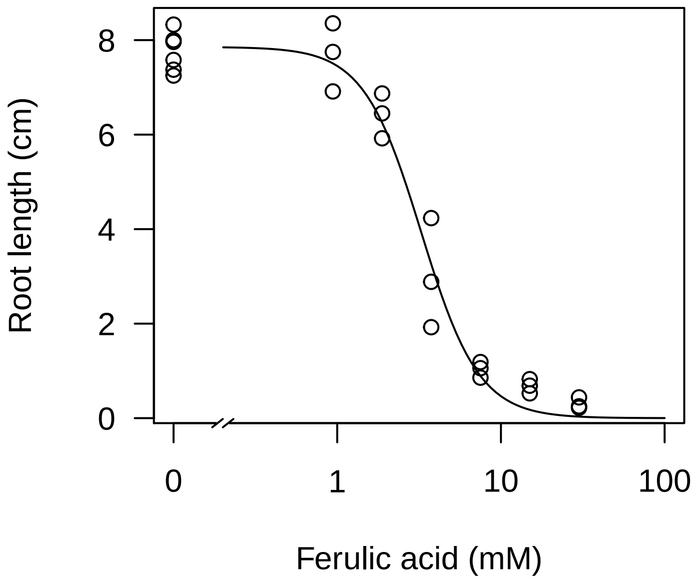

Now we show how functionality of *drc* may provide output for constructing a high-quality plot with the extension package *ggplot2*.

```
# new dose levels as support for the line
newdata <- expand.grid(conc=exp(seq(log(0.5), log(100), length=100)))
# predictions and confidence intervals
pm <- predict(ryegrass.LL.4, newdata=newdata, interval="confidence")
# new data with predictions
newdata$p <- pm[,1]
newdata$pmin <- pm[,2]
newdata$pmax <- pm[,3]

# plot curve
library(ggplot2)
# need to shift conc == 0 a bit up, otherwise there are problems with coord_trans
ryegrass$conc0 <- ryegrass$conc
ryegrass$conc0[ryegrass$conc0 == 0] <- 0.5
# plotting the curve
ggplot(ryegrass, aes(x = conc0, y = rootl)) +
  geom_point() +
  geom_ribbon(data=newdata, aes(x=conc, y=p, ymin=pmin, ymax=pmax), alpha=0.2) +
  geom_line(data=newdata, aes(x=conc, y=p)) +
  coord_trans(x="log") +
  xlab("Ferulic acid (mM)") + ylab("Root length (cm)")
```

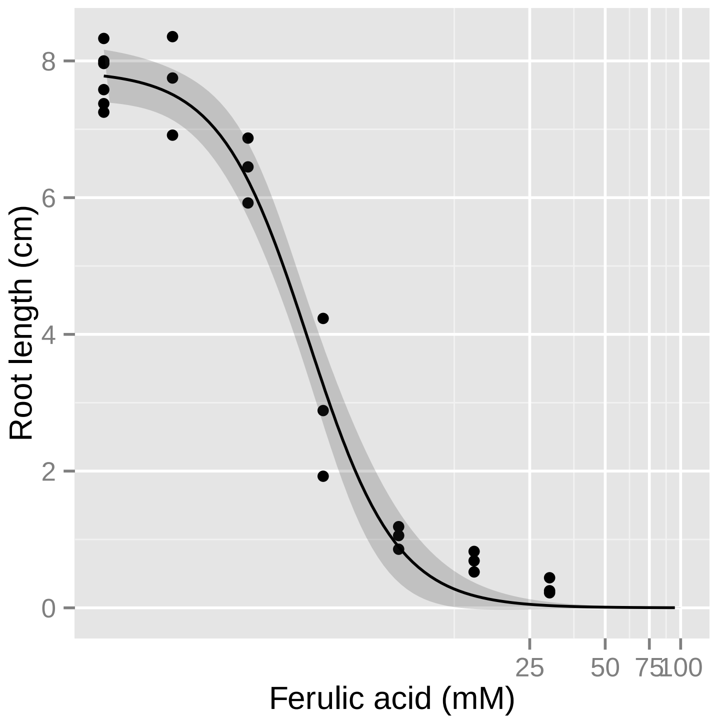

As dose-response analysis is a type of regression analysis it is natural to show confidence bands around the fitted regression curve instead of providing error bars at each dose in the dataset.

Example 3: Continuous response: two dose-response curves

Here is another example involving a continuous response. Data are from an experiment comparing the potency of the two herbicides glyphosate and bentazone in white mustard (*Sinapis alba*) (Christensen *et al.*, 2003). Pay special attention to the use of the argument `pmodels` to incorporate the assumption that the lower and upper limits for the two herbicides are identical, whereas slopes and ED50 parameters are different (in total 6 parameters).

```
S.alba.LL.4.1 <- drm(DryMatter~Dose, Herbicide, data=S.alba, fct = LL.4(),
pmodels=list(~Herbicide-1, ~1, ~1, ~Herbicide-1))
summary(S.alba.LL.4.1)

##
## Model fitted: Log-logistic (ED50 as parameter) (4 parms)
##
## Parameter estimates:
##
##                         Estimate Std. Error   t-value p-value
## b:HerbicideBentazone    5.046141   1.040135  4.851430       0
## b:HerbicideGlyphosate   2.390218   0.495959  4.819387       0
## c:(Intercept)           0.716559   0.089245  8.029117       0
## d:(Intercept)           3.854861   0.076255 50.551925       0
## e:HerbicideBentazone   28.632355   2.038098 14.048566       0
## e:HerbicideGlyphosate  66.890545   5.968819 11.206663       0
##
## Residual standard error:
##
##  0.3705151 (62 degrees of freedom)
```

To demonstrate that the two dose-response curves are not the same we fitted the simpler model not distinguishing between the two curves (in total 4 parameters). Note that for the specification of this model in `drm()` the second argument, which is used to indicate multiple curves, and the argument `pmodels` were omitted. Based on these two model fits we calculated an approximate F-test:

```
S.alba.LL.4.2 <- drm(DryMatter~Dose, data=S.alba, fct = LL.4())
anova(S.alba.LL.4.2, S.alba.LL.4.1)

##
## 1st model
##  fct:     LL.4()
##  pmodels: 1 (for all parameters)
## 2nd model
##  fct:     LL.4()
##  pmodels: ~Herbicide - 1, ~1, ~1, ~Herbicide - 1
## ANOVA table
##
##            ModelDf     RSS Df F value p value
## 1st model       64 20.0153
## 2nd model       62  8.5114  2  41.899   0.000
```

The p-value is below 0.0001, rejecting the null hypothesis that the two slopes and ED50 parameters are the same for the two herbicides and, consequently, rejecting that the two dose-response curves are identical. The estimated relative potency based on the two ED50s is obtained using the function `EDcomp()`:

```
EDcomp(S.alba.LL.4.1, c(10, 50, 50), interval = "delta")

##
## Estimated ratios of effect doses
## (Delta method-based confidence interval(s))
##
##                              Estimate   Lower   Upper
## Bentazone/Glyphosate:10/50  0.27694 0.19936 0.3545
## Bentazone/Glyphosate:10/50  0.27694 0.19936 0.3545
## Bentazone/Glyphosate:50/50  0.42805 0.34026 0.5158
```

Thus, bentazone is approximately half as potent as glyphosate. Comparison of slopes by means of a z-test may be achieved using the function `compParm()` as follows:

```
compParm(S.alba.LL.4.1, "b", "-")

##
## Comparison of parameter 'b'
##
##                                        Estimate Std. Error t-value p-value
## HerbicideBentazone-HerbicideGlyphosate   2.6559     1.0689  2.4847  0.0157
```

Note that this is another way to compare slopes and the p-value is still significant but different from the one we found above using the approximate F test.

Example 4: Generalized nonlinear regression for a binomial response

Earthworm tests are commonly used to evaluate toxicity in ecotoxicology. The built-in dataset `earthworms` in *drc* contains data from such a test. Specifically, the dataset contains the number of earthworms remaining in a container that is contaminated with a toxic substance (not disclosed) instead of migrating to the neighbouring uncontaminated container). For dose 0 a fifty-fifty distribution between the two containers is to be expected. Therefore, we fitted a three-parameter log-logistic model to binomial data. This is an example of a generalized nonlinear model (Finney, 1971). Specifically, the

model is a logistic regression model with a parameter-dependent link function. This model is fitted as follows in **R**:

```
## Fitting an extended logistic regression model
##  where the upper limit is estimated
earthworms.m1 <- drm(number/total ~ dose, weights = total, data = earthworms,
fct = LL.3(), type = "binomial")

summary(earthworms.m1)

##
## Model fitted: Log-logistic (ED50 as parameter) with lower limit at 0 (3 parms)
##
## Parameter estimates:
##
##                Estimate Std. Error  t-value p-value
## b:(Intercept) 1.505679   0.338992 4.441641   0e+00
## d:(Intercept) 0.604929   0.085800 7.050498   0e+00
## e:(Intercept) 0.292428   0.083895 3.485636   5e-04
```

Perhaps even better (more true to the design) it would be fit a log-logistic model where the upper limit is not estimated but instead fixed at 0.5; this is done in the following **R** lines:

```
## Fitting an extended logistic regression model
##  where the upper limit is estimated
earthworms.m2 <- drm(number/total ~ dose, weights = total, data = earthworms,
fct = LL.3(fixed = c(NA, 0.5, NA)), type = "binomial")

summary(earthworms.m2)

##
## Model fitted: Log-logistic (ED50 as parameter) with lower limit at 0 (2 parms)
##
## Parameter estimates:
##
##                Estimate Std. Error  t-value p-value
## b:(Intercept) 1.646689   0.376494 4.373742       0
## e:(Intercept) 0.377269   0.076785 4.913299       0
```

By fixing the upper limit there is a very slight gain in precision for the parameter e, which corresponds to ED50. In contrast, but in this case less interesting, the precision of the slope parameter b is reduced as the dose 0 is important for estimating the slope.

Example 5: Binomial response and four dose-responses curves

Now we considered comparison of four dose-response curves corresponding to four types of selenium (Jeske *et al.*, 2009). First, we fit a (joint) model assuming different ED50 values for the different types of selenium:

```
selenium.LL.2.1 <- drm(dead/total ~ conc, type, weights = total,
data = selenium, fct = LL.2(), type = "binomial")
```

Next, we fitted a model assuming a common ED50 for all four types of selenium and then we compared the two model fits using a likelihood ratio test (a chi-square test):

```
selenium.LL.2.2 <- drm(dead/total~conc, type, weights = total,
data = selenium, fct = LL.2(), type="binomial",
pmodels = list(~factor(type)-1, ~1))

anova(selenium.LL.2.2, selenium.LL.2.1)

##
## 1st model
##  fct:     LL.2()
##  pmodels: ~factor(type) - 1, ~1
## 2nd model
##  fct:     LL.2()
##  pmodels: type (for all parameters)
## ANOVA-like table
##
##           ModelDf  Loglik Df LR value p value
## 1st model       5 -437.99
## 2nd model       8 -376.21  3   123.56       0
```

The four ED50 values are not identical ($p < 0.0001$), which was also concluded previously (Jeske *et al.*, 2009). To quantify differences we calculated unadjusted as well as adjusted 95% confidence intervals (adjustment for simultaneous inference):

```
ED(selenium.LL.2.1, c(50), interval = "delta")

##
## Estimated effective doses
## (Delta method-based confidence interval(s))
##
##        Estimate Std. Error    Lower  Upper
## e:1:50 252.2556    13.8268 225.1555 279.36
## e:2:50 378.4605    39.3707 301.2953 455.63
## e:3:50 119.7132     5.9054 108.1389 131.29
## e:4:50  88.8053     8.6161  71.9180 105.69

library(multcomp)
selenium.EDres <- ED(selenium.LL.2.1, c(50), interval = "delta",
                     multcomp = TRUE, display = FALSE)
confint(glht(selenium.EDres[["EDmultcomp"]]))

##
##   Simultaneous Confidence Intervals
##
## Fit: NULL
##
## Quantile = 2.4909
## 95% family-wise confidence level
##
##
## Linear Hypotheses:
```

```
##              Estimate lwr      upr
## e:1:50 == 0 252.2556 217.8141 286.6970
## e:2:50 == 0 378.4605 280.3913 476.5297
## e:3:50 == 0 119.7132 105.0034 134.4230
## e:4:50 == 0  88.8053  67.3432 110.2674
```

The adjusted confidence intervals become slightly wider as compared to the unadjusted ones.

References

Christensen, M. G., Teicher, H. B. & Streibig, J. C. (2003). Linking fluorescence induction curve and biomass in herbicide screening. *Pest Management Science* **59**, 1303–1310.

Environment Canada (2005). *Guidance Document on Statistical Methods for Environmental Toxicity Tests*. Environment Canada, Ottawa.

Finney, D. J. (1971). *Probit Analysis*. Cambridge University Press, London, third edn.

Inderjit, Streibig, J. C. & Olofsdotter, M. (2002). Joint action of phenolic acid mixtures and its significance in allelopathy research. *Physiologia Plantarum* **114**, 422–428.

Jeske, D. R., Xu, H. K., Blessinger, T., Jensen, P. & Trumble, J. (2009). Testing for the Equality of EC50 Values in the Presence of Unequal Slopes With Application to Toxicity of Selenium Types. *Journal of Agricultural, Biological, and Environmental Statistics* **14**, pp. 469–483. URL `http://www.jstor.org/stable/20696589`.

Stephan, C. E. (1977). Methods for Calculating an LC_{50}. In *Aquatic Toxicology and Hazard Evaluation* (eds. F. L. Mayer & J. L. Hamelink), ASTM STP 634, 65–84. American Society for Testing and Materials.
